# Association between Abnormal Echocardiography and Adverse Obstetric Outcomes in Low-Risk Pregnant Women

**DOI:** 10.3390/jcdd9110394

**Published:** 2022-11-15

**Authors:** Kerrilynn C. Hennessey, Thara S. Ali, Eunjung Choi, Alexandra R. Ortengren, Leigh C. Hickerson, Jennifer May Lee, Cynthia C. Taub

**Affiliations:** 1 Section of Cardiovascular Medicine, Heart and Vascular Center, Dartmouth-Hitchcock Health System, Lebanon, NH 03756, USA; 2 Division of Cardiology, Johns Hopkins University School of Medicine, Baltimore, MD 21287, USA; 3 RecoverX, London W4 1NG, UK

**Keywords:** pregnancy, echocardiography, obstetric outcomes, pre-term delivery, caesarean section

## Abstract

Maternal mortality in the United States is a public health crisis of preventable deaths among young women. The role of echocardiography in the evaluation of pregnant women with cardiovascular symptoms or risk factors without known heart disease is unclear. We retrospectively examined the clinical characteristics of consecutive pregnant patients without established heart disease who underwent echocardiography and evaluated associations between abnormal exam findings and obstetric outcomes. Among low-risk women undergoing echocardiography during pregnancy, older age, higher parity and a history of chronic hypertension are associated with a higher likelihood of echocardiographic abnormalities, which in turn are associated with a higher likelihood of adverse obstetric outcomes including caesarean section and preterm delivery.

## 1. Introduction

Identifying clinical predictors for adverse cardiovascular and obstetric outcomes in pregnant women is a major public health priority. Maternal mortality in the United States has been increasing over the past two decades and is currently 50% higher than Western Europe [1]. The rise in maternal mortality has been attributed to an increasing number of pregnant women with advanced maternal age, obesity, diabetes mellitus, hypertension and underlying congenital heart disease [2]. Racial and geographic disparities in adverse pregnancy outcomes are most pronounced in urban settings [3]. Black women are particularly at high risk, with a 3- to 4-fold increased risk of death due to pregnancy complications compared to their white counterparts [4]. While these disparities are also present in rural counties, it is imperative that close attention is directed toward maternal health in racially diverse urban regions.

Established risk stratification indices such as ZAHARA [5], mWHO [6], and CARPREG II [7] are widely used to predict adverse cardiovascular outcomes in women with congenital heart disease. Although obstetric outcomes were not part of the primary endpoints in these studies [5,6,7], high rates of obstetric (both maternal and fetal) complications have been reported in women with known congenital heart disease in subsequent studies [8,9]. In the general population, hypertensive disorders of pregnancy with and without preeclampsia have been associated with adverse obstetric outcomes including preterm delivery and stillbirth [10,11]. Other clinical characteristics associated with adverse obstetric outcomes are advanced maternal age, body mass index > 40, and twin pregnancy [12,13,14]. There is little research on how these clinical characteristics predict presence of structural heart disease and subsequent obstetric complications in relatively low risk women (i.e., those without known congenital heart disease or high risk features defined in the CARPREG II risk prediction index).

Echocardiography serves as a safe, non-invasive diagnostic tool for identification of cardiac abnormalities in pregnancy. The American College of Obstetrics and Gynecology recommends transthoracic echocardiography be performed in all pregnant women with structural heart disease, congenital heart disease, pulmonary hypertension, or a history of exposure to cardiotoxic chemotherapeutic agents [15]. However, there is insufficient information about the role of echocardiography in pregnant women without known structural heart disease and whether abnormal echocardiographic findings predict worse obstetric outcomes in this population. A recent single center study by Schnettler et al. found that clinical features such as postpartum status, multiparity and tobacco use were associated with abnormal echocardiographic findings in a predominantly white cohort (63% white vs. 28% black) [16]. While they did not find any association with adverse obstetric outcomes, they reported that abnormal echocardiographic findings led to change in antepartum management.

In our study, we aim to examine the role of echocardiography in pregnant women without known high risk structural heart disease, hereafter referred to as low-risk pregnant women. Our primary objective is to identify patient characteristics associated with echocardiographic abnormalities and adverse obstetric outcomes in a racially diverse urban population.

## 2. Materials and Methods

We retrospectively examined 254 consecutive pregnant patients who underwent echocardiography at the Montefiore Medical Center in the Bronx, New York between January 2008 and August 2011. The study received approval from the local research ethics board and consent was waived due to the retrospective nature of the study. Each patient underwent comprehensive echocardiography according to the standard International Commission of Accreditation of Echocardiography Laboratories protocol using Philips iE33 ultrasound system (Philips Medical Systems, Andover, MA, USA). Echocardiographic exams were read and reported by cardiovascular physicians certified in echocardiography by the National Board of Medical Examiners. Echocardiograms were not over-read for the purposes of this study. We excluded patients who had any risk factors for adverse cardiac outcomes in pregnancy, as identified in CARPREG II [7]: at least mild reduction in systemic ventricular systolic function (ejection fraction < 55%), high-risk valve lesions/left ventricular outflow tract obstruction (aortic valve area < 1.5 cm^2^, subaortic gradient > 30 mm Hg, mitral valve area < 2 cm, or moderate to severe mitral regurgitation), mechanical valves, pulmonary hypertension (right ventricular systolic pressure greater than or equal to 50 mm Hg in the absence of right ventricular outflow obstruction), high-risk aortopathy (ex. Marfan syndrome, bicuspid aortopathy with aortic dimension > 45 mm, or prior aortic dissection), coronary artery disease (defined as angiographically proven coronary obstruction or past myocardial infarction) and any prior cardiac intervention (repair of cardiac congenital lesions, valvular replacements or repairs, or percutaneous/operative treatment of arrhythmias). Repeat exams for follow-up during pregnancy were excluded.

Chart review was performed to identify the indication for echocardiogram, patient characteristics and echocardiographic findings. Echocardiographic reports were categorized as abnormal or normal based on the following guidelines: Echocardiograms were classified as normal if (1) Biventricular size, biventricular systolic function, left ventricular diastolic function, biatrial size, and thoracic aorta size were normal based on American Society of Echocardiography reference ranges for cardiac chamber quantification published in 2015 [17]. For the purposes of this study, left ventricular hypertrophy and atrial dilatation were only considered abnormal if they were moderate or severe. (2) Valvular regurgitation was mild or less and there was no valvular stenosis according to ASE guidelines for the assessment of valve stenosis and regurgitation published in 2009 and 2003, respectively [18,19]. (3) Pulmonary artery systolic pressure (PASP) was considered normal when less than 40 mmHg to improve specificity for potentially hemodynamically significant pulmonary hypertension given the expected increase in blood volume in pregnancy may lead to overestimation of this flow dependent measure. If a study did not meet full criteria for a normal exam, it was categorized as abnormal with the qualifying abnormality noted. Pregnancy outcomes were defined according to the American College of Obstetricians and Gynecologists.

Variables were organized into categories and expressed as percentages. We categorized obesity by BMI groups, with class I obesity defined as BMI 30 to <35, class II as BMI 35 to <40, and class III obesity as BMI 40 or higher. As the BMI data available were the most recent height and weight recorded in the medical record immediately prior to the echocardiography exam and over 90% of exams were performed in the second or third trimester, a BMI of >40 was used to improve the specificity for pregnant women with at least class I pre-gravid obesity for the analysis. These categorical variables were then compared using Chi-Square tests. A *p*-value less than 0.05 was considered statistically significant.

## 3. Results

Among 254 consecutive pregnant women who underwent echocardiography, 195 met inclusion criteria. Clinical characteristics of these patients are demonstrated in Table 1. This is a racially diverse population with over 40% self- identifying as black and over 30% identifying as multi-racial. Echocardiograms were ordered for pregnant women for a wide range of indications, as demonstrated in Table 2. Symptom driven indications included chest pain, shortness of breath and palpitations. Echocardiograms were also ordered for abnormal physical exam findings, history of an abnormal ECG or arrhythmia, history of cardiac disease or syncope, suspected cardiac disease and in one case, twin gestation. The most common indication for an echocardiogram among low-risk pregnant women was to evaluate a murmur.

**Figure 1:**
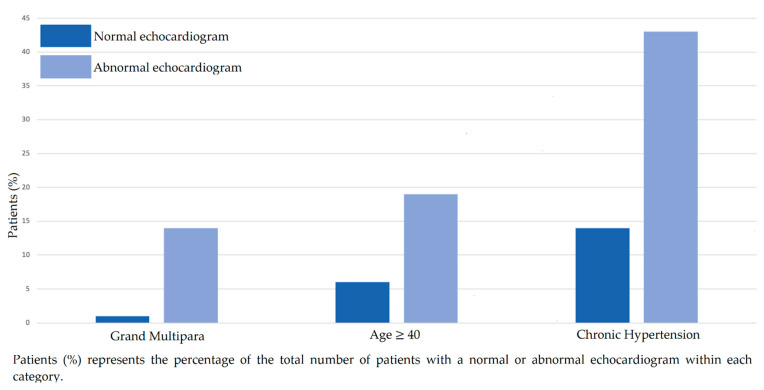
Percentage of normal and abnormal echocardiograms by grand multipara, age, and hypertensive status.

A total of 21 echocardiograms (11%) were identified as abnormal. (Table 3). Pregnant women with chronic hypertension were more likely to have an abnormal echocardiogram compared to those without hypertension (*p* < 0.01) (Table 4). Older age and higher parity were also associated with an abnormal echocardiogram (*p* = 0.04 and *p* < 0.01, respectively). There was no association between race, BMI, gestational age or any other medical comorbidity (including diabetes) and abnormal findings. There was no association between any other indication for echocardiogram and an abnormal result. The most common abnormal finding was elevated PASP. Figure 1 demonstrates representative comparisons between the proportion of patients with normal and abnormal echocardiograms and older age, higher parity and a history of chronic hypertension.

**Figure 2:**
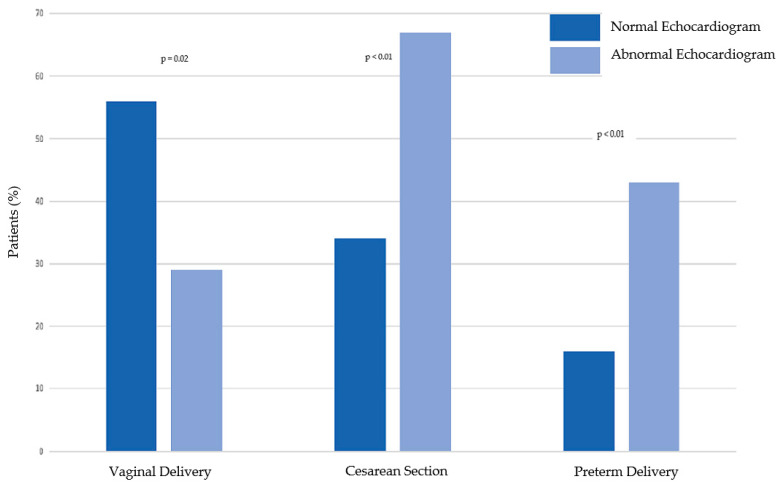
Obestetric outcomes among low-risk pregnant women who undergo echocardiography.

The majority of low-risk pregnant women undergoing echocardiography had a vaginal delivery (53%) (Table 5). Thirty-eight percent underwent Cesarean section and 19% had preterm labor. A comparison of pregnancy outcomes among patients with normal and abnormal echocardiograms is shown in Table 5 and graphically in Figure 2. Low-risk pregnant women with abnormal echocardiograms are significantly more likely to undergo Cesarean section and have a preterm delivery than those with normal echocardiograms (*p* < 0.01 for each). In a post hoc analysis, the study is powered at 79.7% with an alpha of 0.05 for the out-come of preterm delivery.

## 4. Discussion

Currently few expert consensus statements are available to guide the use of echocardiography in pregnant women without known cardiac disease. This analysis sought to identify patient characteristics associated with abnormal echocardiography results and obstetric outcomes in a racially diverse population of relatively low risk pregnant women to better understand the role of echocardiography on clinical management. We found that approximately 10% of initial echocardiography requests for low risk pregnant women had abnormal findings, regardless of study indication. Women with higher parity and those with chronic hypertension were more likely to have an abnormal echocardiogram. Abnormal echocardiography results were associated with adverse obstetric outcomes including preterm delivery and a caesarean mode of delivery.

Several studies have demonstrated that the appropriateness criteria for echocardiography are applicable in pregnancy, however, the rate of normal results is high, even in exams with appropriate indications [16,20]. Consideration of clinical risk factors such as multiparity and tobacco use was suggested by Schnettler et al. to improve the likelihood of finding clinically significant abnormalities. Our study confirms the finding of Schnettler et al. that increasing parity is associated with abnormal echocardiographic findings during pregnancy. This finding persists when adjusting for age. The rate of abnormal echocardiograms in our study was lower than the Schnettler cohort (11% vs. 36%), which likely reflects our decision to exclude women with identified risk factors based on the CARPREGII risk score and define a PASP threshold of 40 mmHg as abnormal.

The association between high-risk clinical characteristics such as diabetes, hypertension, and class III obesity and echocardiographic abnormalities has recently been suggested by Hopkins et al. [21]. In women with class III obesity pre-pregnancy and comorbid conditions, 40% had an abnormal screening echocardiogram, which was in turn associated with preterm delivery. BMI > 40 recorded at or around the time of echocardiography was not statistically associated with abnormal findings on echocardiography in our cohort, but it is possible the pre-pregnancy assessment for obesity used by Hopkins et al. is a better indicator of cardiometabolic risk. We add that chronic hypertension is associated with abnormal echocardiographic findings, even in the absence of class III obesity. In our study, almost 50% of women with an abnormal echocardiogram carried a diagnosis of chronic hypertension. Echocardiographic abnormalities in pregnant women with hypertension included evidence of left ventricular pressure overload including moderate or severe hypertrophy, diastolic dysfunction, and pulmonary hypertension defined as pressures greater than 40 mmHg.

Elevated PASP of greater than 40 mmHg was the most common echocardiographic abnormality in our study accounting for almost 50% of abnormal echocardiograms. The cut-off for diagnosing pulmonary hypertension using non-invasively estimated PASP in pregnancy is not well established. In one prior report looking at young pregnant women with established cardiovascular disease almost one-third of patients with pulmonary hypertension defined as pulmonary artery systolic pressure greater than 30 mmHg had normal pulmonary pressures by invasive catheterization [22]. Measurement of pulmonary pressure using the Bernoulli equation is problematic during pregnancy because this is a flow-derived measure and pregnancy can increase cardiac output by up to 40% [23]. Noninvasive estimates of right atrial pressure using IVC size similarly do not account for the increased blood volume and decreased SVR of pregnancy which may contribute to an overestimation of PASP. A cut-off of 40 mmHg was used in our study to increase the specificity of non-invasively derived PASP for potential pathology, however, an increased risk of pre-term delivery was present even when a lower threshold of 35 mmHg was analyzed. An additional 11 patients would have been classified as abnormal using the normal cutoff value of 35 mmHg, increasing the rate of abnormal echocardiograms to 16% in this cohort [24]. This suggests that even mild elevations in pulmonary pressures in response to pregnancy may have physiologic significance and additional study of PASP thresholds and outcomes are warranted.

In addition to corroborating clinical characteristics which have been reported to be associated with abnormal echocardiography results during pregnancy and categorizing the abnormalities, our work found that echocardiographic findings are associated with adverse obstetric outcomes. Previous studies reported conflicting evidence with regard to the association between abnormal echocardiography in pregnancy and adverse obstetric outcomes. Schnettler et al. did not find an association between abnormal echocardiography results and obstetric outcomes [16]. Hopkins et al. found an association between abnormal echocardiography results and pre-term delivery in a population of largely black (90%) women with class III obesity [21]. Among women with abnormal echocardiograms in our cohort of racially diverse women both with and without class III obesity, we found that more than 40% delivered prematurely. In fact, abnormal echocardiography results were associated with a similar risk of preterm delivery as a history of prior preterm birth or miscarriage, which is the strongest known clinical predictor of pre-term delivery [25]. Preterm birth is a serious obstetric problem. It is the most common cause of infant death and is the leading cause of long-term disability related to the nervous system in children [26,27,28]. Risk stratification for preterm birth is incredibly important to guide antenatal management including prophylactic interventions. Chronic hypertension is known increase the risk of preterm birth by 1.5 to 4 times, and abnormalities on echocardiogram may further increase this risk. The four twin gestations among our cohort all had normal echocardiograms suggesting that the association between abnormal findings and preterm delivery might not be mediated through multiple gestations. However, the sample size for twin pregnancy was too small to draw meaningful conclusion.

To our knowledge, this is the most racially diverse study to examine the association between clinical comorbidities, abnormal echocardiography results, and adverse obstetric outcomes among low risk pregnant women. While self-identified black race has been identified as a risk factor for adverse maternal and fetal outcomes in the United States, our study did not find a statistically significant difference in the detection of abnormalities by echocardiography or obstetric outcomes by race when accounting for patient age, parity, and the presence of chronic hypertension. This may reflect the fact that 70% of women in our cohort identified as black or multiracial and the number of Caucasian women was too low to uncover disparities.

### Limitations

This study has several limitations related to its retrospective design that must be taken into consideration. First, echocardiographic and clinical parameters were abstracted from the medical record, limiting the data collected to that which is charted. While adequately powered for the present analysis, our sample size is relatively small. However, our study collected data on consecutive echocardiograms performed on pregnant individuals, which represented a meaningfully substantial cohort. The BMI data available were the most recent height and weight recorded in the medical record immediately prior to the echocardiography exam, therefore, they may not accurately capture the co-morbidity of obesity. BMI > 40 was defined as obese as women with class III obesity during pregnancy were likely to have at least class I pre-gravid obesity. The study indication and echocardiographic findings were similarly determined from the original clinical reports without over-reading. This limitation is mitigated by the fact that this study was performed through an IAC accredited echocardiography laboratory where all readers are board certified. The outcome of pre-term birth was not further classified as spontaneous or medically indicated, therefore, it is difficult to assess if pre-term birth was a social-structural reaction to an abnormal echocardiogram or if it reflects a biologic predisposition to early labor. Finally, the decision to perform echocardiography on these pregnant patients may reflect a selection bias, however, study indications suggest the exams were appropriate based on criteria used in clinical practice.

## 5. Conclusions

Among low-risk women undergoing echocardiography during pregnancy, older age, higher parity and a history of chronic hypertension are associated with a higher likelihood of echocardiographic abnormalities, which in turn are associated with a higher likelihood of Cesarean section and preterm delivery. The role of abnormal echocardiographic findings in the antenatal management of hypertensive women warrants further investigation with specific attention to spontaneous vs. medically indicated preterm birth.

## Figures and Tables

**Table 1:** Baseline characteristics of low-risk pregnant women who underwent echocardiography.

Variable, No. (%)		
N	**195**	
Twin gestation	4	(2)
Parity status		
Nulliparous	66	(34)
Primiparous	60	(31)
Parity of 2 to 5	56	(29)
Grand Multipara	5	(3)
Unknown	8	(4)
Race		
African American	79	(41)
Multi-racial	60	(31)
White	16	(8)
Asian	2	(1)
Unidentified	33	(17)
Age, y		
<20	6	(3)
20–24	34	(18)
25 to 29	55	(28)
30 to 34	46	(24)
35 to 39	39	(20)
≥40	15	(8)
Obesity		
Not obese	87	(45)
Class I	44	(23)
Class II	28	(14)
Class III	29	(15)
Unknown	7	(4)
Comorbidities		
Arrhythmia	5	(3)
Hypertension	34	(17)
Diabetes	23	(12)
Anemia	16	(8)
Sickle Cell Disease	10	(5)
Connective Tissue Disease	14	(7)
HIV *	6	(3)
Pulmonary Embolism	6	(3)
Cerebrovascular Event	3	(2)
Gestational Age		
1st Trimester	13	(7)
2nd Trimester	108	(55)
3rd Trimester	71	(36)
Unknown	3	(2)

* HIV = human immunodeficiency virus.

**Table 2:** Indications for echocardiography among low-risk pregnant women.

	Population Total		Normal Echocardiogram		Abnormal Echocardiogram		*p* Value
Variable, No. (%)							
N	195		174		21		
Indication for Echocardiogram							
Shortness of breath	40	(21)	37	(21)	3	(14)	0.45
Syncope	18	(9)	17	(10)	1	(5)	0.56
Chest pain	10	(5)	9	(5)	1	(5)	0.94
Palpitations	23	(12)	22	(13)	1	(5)	0.29
Murmur	44	(23)	39	(22)	5	(24)	0.89
Abnormal ECG or arrhythmia	19	(10)	19	(11)	0	(0)	
History of HTN *	12	(6)	5	(3)	7	(33)	<0.01
History cardiac disease or pHTN ^+^	15	(8)	13	(7)	2	(10)	0.74
Suspected cardiac disease or pHTN ^+^	12	(6)	11	(6)	1	(5)	0.78
Twin pregnancy	1	(1)	1	(1)	0	(0)	
Unknown	1	(1)	1	(1)	0	(0)	

* HTN = chronic or gestational hypertension; ^+^ pHTN = pulmonary hypertension.

**Table 3:** Abnormal findings on echocardiography among low-risk pregnant women.

Variable, No. (%)		
Total Abnormal	21	(11)
Abnormal Findings (% of Total Abnormal)		
Pulmonary artery systolic pressure ≥ 40 mmHg	10	(48)
Left ventricular regional wall motion abnormality	3	(14)
Left ventricular diastolic dysfunction (impaired relaxation)	2	(10)
Right ventricular systolic dysfunction	3	(14)
Left ventricular hypertrophy (>1.2 cm thickness)	2	(10)
Valve disease (more than mild mitral regurgitation, any stenosis)	3	(14)
Left atrial dilatation (more than mild)	3	(14)

**Table 4:** Characteristics of low-risk pregnant women with normal and abnormal echocardiograms.

	Normal Echocardiogram		Abnormal Echocardiogram		*p* Value
Variable, No. (%)					
N	174		21		
Twin gestation	4		0		
Parity status					<0.01
Nulliparous	60	(34)	6	(29)	
Primiparous	54	(31)	6	(29)	
Parity of 2 to 5	51	(29)	5	(24)	
Grand Multipara	2	(1)	3	(14)	
Unknown	7	(4)	1	(5)	
Race					
African American	68	(39)	11	(52)	0.24
Multi-racial	57	(33)	5	(24)	0.41
White	14	(8)	2	(10)	0.82
Asian	2	(1)	0	(0)	
Unidentified	33	(19)	3	(14)	
Age, y					0.04
<20	6	(3)	0	(0)	
20–24	28	(16)	6	(29)	
25 to 29	53	(30)	2	(10)	
30 to 34	43	(25)	3	(14)	
35 to 39	33	(19)	6	(29)	
≥40	11	(6)	4	(19)	
Obesity					0.09
Not obese	79	(45)	8	(38)	
Class I	43	(25)	1	(5)	
Class II	23	(13)	5	(24)	
Class III	24	(14)	5	(24)	
Unknown	5	(3)	2	(10)	
Comorbidities					
Arrhythmia	4	(2)	0	(0)	
Hypertension	25	(14)	9	(43)	<0.01
Diabetes	19	(11)	4	(19)	0.28
Anemia	14	(8)	2	(10)	0.82
Sickle Cell Disease	10	(6)	3	(14)	0.14
Connective Tissue Disease	11	(6)	3	(14)	0.18
HIV *	5	(3)	1	(5)	0.64
Pulmonary Embolism	6	(3)	0	(0)	
Cerebrovascular Event	2	(1)	1	(5)	0.20
Gestational Age					0.60
1st Trimester	13	(7)	0	(0)	
2nd Trimester	97	(56)	11	(52)	
3rd Trimester	62	(36)	9	(43)	
Unknown	2	(1)	1	(5)	

* HIV = human immunodeficiency virus

**Table 5:** Obstetric outcomes among low-risk pregnant women by echocardiography result.

	Population Total		Normal Echocardiogram		Abnormal Echocardiogram		*p* Value
Variable, No. (%)							
N	195		174		21		
Outcome of Delivery							
Vaginal Delivery	104	(53)	98	(56)	6	(29)	0.02
Cesarean Section	74	(38)	60	(34)	14	(67)	<0.01
Pregnancy Outcomes							
Preterm Delivery	37	(19)	27	(16)	9	(43)	<0.01
Spontaneous Abortion	2	(1)	2	(1)	0	(0)	
Intra-Uterine Fetal Demise	2	(1)	2	(1)	0	(0)	

## Data Availability

Not applicable.
